# The Effect of Various Stabilizers on Preserving Immunogenicity of Lyophilized Mumps Vaccines

**Published:** 2017-09-12

**Authors:** Razieh Kamali-Jamil, Mohammad Shayestehpour, Zohreh-Azita Sadigh, Mohammad Taqavian, Mohammad-Kazem Shahkarami, Fatemeh Esna-Ashari, Reza Shahbazi, Ashraf Mohammadi, Abolhasan Foroughi, Bizhan Romani

**Affiliations:** ^1^ Human Viral Vaccine Department, Razi Vaccine & Serum Research Institute, Agricultural Research, Education & Extension Organization (AREEO), Karaj, Iran; ^2^ Department of Biochemistry, University of Alberta, Edmonton, Alberta, Canada; ^3^ Cellular and Molecular Research Center, Faculty of Medicine, Ahvaz Jundishapur University of Medical Sciences, Ahvaz, Iran

**Keywords:** Immunogenicity, Vaccine, Mumps Virus

## Abstract

**Background:** Chemical stabilizers are added to live attenuated vaccines for enhancing the virus stability. The aim of this study was to evaluate the effect of various stabilizers on preserving immunogenicity of lyophilized mumps vaccines.

**Study design:** An experimental study.

**Methods:** Three mumps vaccines with different formulations were inoculated to three groups of Guinea pigs. Sterile water was injected to eight Guinea pigs as a control group. Blood samples were collected before inoculation and on 14, 28 and 42 d after vaccine injection. Mumps antibodies in the sera were measured using hemagglutination inhibition assay (HAI).

**Results:** All three formulated mumps vaccines induced antibody in Guinea pigs after two weeks. Formulation 1 containing trehalose dihydrate and formulation 2 comprised human serum albumin stimulated antibodies in the higher level than Razi routine formulation.

Conclusions: Various stabilizers have different preservation potencies that differently affect immune response against virus. More stable and more immunogenic vaccines can be produced using stabilizers containing trehalose dihydrate.

## Introduction

Chemical stabilizers are added to live attenuated vaccines for enhancing the virus stability^1^. Main ingredients of these stabilizers are hydrolyzed gelatin, sucrose, sorbitol, and other sugars^2, 3^. Stabilizing components can serve as antigen preservatives and increase antibody response to vaccine. For example, application of trehalose in vaccine formulation stimulates a strong immune response^4^. The efficacy of live vaccines depends on their ability for immune stimulation and their thermal stability^2, 5^. Changing vaccine formulation and increasing vaccine-induced immune response can be applied for production of a more effective vaccine.

Mumps is a viral disease caused by a paramyxovirus responsible for serious health problems, such as meningitis, encephalitis, and infertility^6^.Vaccination is the best approach to prevent this infection^7^. Immunization against mumps is recommended to all people of 12 months or older^8^. Mumps inactivated vaccines are not administrated anymore since they have disadvantages, including requirement for additional dose injection, delay in cellular immunity stimulation and unstable protection ^6^. Today, live attenuated mumps strains combined with rubella and measles viruses (MMR) produce long-lasting immunity and therefore they are used for vaccination ^9, 10^. Up to date, more than ten attenuated strains of mumps virus have been used for vaccine production^8^. The RS-12 strain of mumps induces a stronger humoral immune response compared to other strains ^11, 12^. Impact of vaccine stabilizers on induction of antibody response in mumps virus (RS12 strain) has not been well addressed.

In this study, we evaluated the effect of various mumps vaccine formulations of RS-12 strain on producing antibodies in Guinea pigs.

## Methods

### Mumps vaccine production

We formulated three lyophilized mumps vaccines using the Iranian RS-12 strain and different stabilizers in Razi Vaccine and Serum Research Institute, Karaj, Iran. The formulation and vaccine production process was previously described ^2^. Formulation 1 contained 0.3 M trehalose dihydrate, 111 mg/ml hydrolyzed gelatin, 8.8 mM KH_2_PO_4_, 0.5 M sodium glutamate and 40 mM Na_2_HPO_4_ in water. In formulation 2, we used 1.24 mg/ml human serum albumin, 3.6 mM sucrose, 385 mM sorbitol, 75 mM Na_2_HPO_4_, 40.2 mg/ml hydrolyzed gelatin, 10 mM sodium bicarbonate and 87 mM NaCl in water. Formulation 3 contained a gelatin-based stabilizer was provided by the Razi Vaccine and Serum Research Institute. All stabilizers were filter sterilized and mixed 1:1 with the harvested mumps virus. Vaccines were lyophilized using the standard lyophilization procedure.

The study was approved by the Ethics Committee of Razi Vaccine and Serum Research Institute, Alborz, Iran.

### Vaccine injection to Guinea pigs and blood collection

We provided 26 male Guinea pigs (Per Bright short hair), weighing 250-350gr, from the Department of Laboratory Animals in Razi Institute of Iran, Karaj, Iran. Animals were divided into four groups. Three groups, each comprising 6 subjects, were allocated for inoculation of three different vaccine formulations and one group (8 Guinea pigs) was used as control. Five hundred microliter of each reconstituted mumps vaccine (only DMEM for the control group) was injected subcutaneously in the animal's right flank. A booster dose was injected to animals after two weeks. Blood samples were collected before inoculation and on 14, 28 and 42 d after vaccine injection.

### Hemagglutination Inhibition Assay (HAI test)

Mumps hemagglutination (HA) antigen with titer 1:32 was obtained from Razi Institute of Iran. Mumps antigen titration was performed using hemagglutination test and Guinea pig red blood cells in two-fold serial dilutions for providing 4-unit mumps antigen. Serum samples were inactivated at 56 °C for 30 min and treated with 25% kaolin. The hemagglutination inhibition (HAI) assay was carried out in V-bottom 96 well plates. Twenty-five microliters of PBS containing 1% albumin were added to each well. Then, 25 ul of 2-fold serial dilutions of the serum samples and 25 µl of 4-unit mumps antigen was added to all wells. Micro plates were shaken and incubated for 1 h at 37 °C. Then, 50 ul of Guinea pig RBC (0.5%) was added to all wells and plates were incubated for 2 h at 37 °C with shaking. All wells were observed for agglutination. A jagged shield of red blood cells or an irregular button indicates agglutination. The HAI titer of each serum sample was the inverse of the last dilution where cells were not agglutinated.

### Statistical Analysis

Student’s 𝑡-test was used to determine significant differences of the data between two groups by GraphPad Prism 6.0 software. A *P* value of lower than 0.05 was considered statistically significant.

## Results

**Figure 1:**
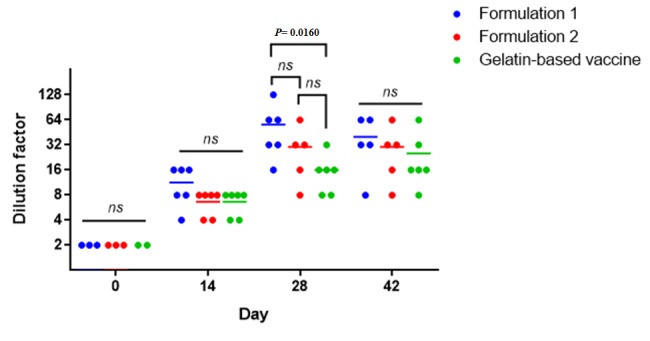
HAI assay for immunization of Guinea pigs against mumps virus. Guinea pigs were vaccinated with 3 different vaccine formulations. Blood samples were collected on day 0, 14, 28, and 42 and their antibody titers against mumps virus were measured using HAI assays.

Twenty-six Guinea pigs serum samples were treated and tested by HAI technique for measuring mumps antibody. In the control group, we did not observe a significant antibody titer (data not shown). The antibody titer on day 14 post-vaccination, before booster inoculation, for vaccine formulation 2 and gelatin-based vaccine was very similar. HAI assay results summarized in Figure 1. Titer of mumps antibody for vaccine formulation 1 was slightly higher than that of the gelatin-based vaccine and formulation 2; however, this was not statistically significant. On 28-d post-vaccination, the difference between formulation 1 and the other vaccine formulations became greater so that formulation 1 had stimulated a significantly higher antibody titer compared to the gelatin-based vaccine. On day 42 post-vaccination, the antibody titer was reduced so that no significant difference was observed among the tested subjects.

## Discussion

Previous studies have used laboratory animals such as rat, monkey, hamster, and Guinea pig to evaluate immune response against different strains of mumps virus ^11-13^. In the present study, using the RS-12 strain, all HAI tests were positive 14 d post-vaccination. Shramek et al. developed an attenuated mumps vaccine with Barnes, Ricki and pomanti strains administrated to Guinea pigs by either aerosol inhalation or a subcutaneous injection^14^. Similarly, to the present study, HAI tests were positive for the attenuated strains after two weeks. In another study, the immune response in mice immunized was compared with four attenuated strains of mumps virus, including Urabe, Jeryl Lynn, Rubini, and RS-12. Interestingly, the RS-12 strain induced a better immunization compared to the other strains^12^. In the present study, all vaccine formulations containing this strain induced efficient immune responses 4 wk postinfection.

Up to date, no published data are available for the effect of various mumps vaccine formulations on stimulating immune response in animals or humans. We found that trehalose dihydrate as stabilizer in formulation 1 induced higher antibody response among three tested vaccine formulations. Addition of trehalose to the influenza vaccine formulation significantly improved preservation of HA activity of viral antigen after drying ^4^. Trehalose was retained immunogenicity of the virus particles in vaccine after drying process. The use of trehalose as stabilizer significantly improves the stability of measles, mumps and rubella vaccines^2, 3, 13^. Trehalose maintained a higher viral titer than human serum albumin and the gelatin-based formulation after freeze drying procedure. Our findings support the previous results for trehalose by showing the best immunogenicity for formulation 1. Formulation 2 contained human serum albumin as stabilizer. Previous studies have demonstrated the role of HSA in virus stability for vaccine production^15, 16^. In the present study, formulation 2 stimulated immune response to an extent that was not significantly higher than the stimulation by gelatin-based formulation.

The limitation of this study was the death of some Guinea pigs before the last blood sampling.

## Conclusions

Various stabilizers have different preservation potencies that differently affect immune response against virus. More stable and more immunogenic vaccines could be produced using different stabilizers such as trehalose dihydrate. We recommend comparing the immunogenicity of three different mumps-containing formulations in human.

## Conflict of interest statement

The authors declare that there is no conflict of interest.

## Funding

Financial support for this research has been provided by Razi Vaccine and Serum Research Institute.

## Highlights

- Three formulated mumps vaccines induced antibody in Guinea pigs after two weeks.
- Various stabilizers had different potency to preserve immunogenicity of mumps virus.
- Trehalose dihydrate as stabilizer induced higher antibody response.

## References

[1] Shahkarami MK, Shayestehpour M, Sancholi A, Taqavian M, Jamil RK, Esna-Ashari F (2015). Application of Real-Time PCR method for evaluation of measles vaccine heat stability. J Paramed Sci.

[2] Jamil RK, Taqavian M, Sadigh ZA, Shahkarami MK, Esna-Ashari F, Hamkar R (2014). Evaluation of the thermal stability of a novel strain of live-attenuated mumps vaccine (RS-12 strain) lyophilized in different stabilizers. J Virol Methods.

[3] Shayestehpour M, Shahkarami MK, Shafyi A, Taqavian M, Kamali jamil R, Esna-ashari F (2012). A study of the thermal stability of measles vaccine produced by AIK-C strain. J Arak Univ Med Sci.

[4] Kim YC, Quan FS, Song JM, Vunnava A, Yoo DG, Park KM (2010). Influenza immunization with trehalose-stabilized virus-like particle vaccine using microneedles. Procedia Vaccinol.

[5] Soleimani S (2016). Stability Study of Measles AIK-C Strain, Mumps RS-12 Strain and Rubella Takahashi Strain in MMR Vaccine. Arch Razi Inst.

[6] Fields BN, Knipe DM, Howley PM. Fields virology. 6th ed. Philadelphia: Wolters Kluwer Health/Lippincott Williams & Wilkins; 2013.

[7] Hamade A, Salameh P, Medlej-Hashim M, Hajj-Moussa E, Saadallah-Zeidan N, Rizk F (2013). Autism in children and correlates in Lebanon: a pilot case-control study. J Res Health Sci.

[8] Plotkin SA, Orenstein WA, Offit PA. Vaccines. 6th ed. Philadelphia: Elsevier; 2013.

[9] Rejali M, Mohammadbeigi A, Mokhtari M, Zahraei SM, Eshrati B (2015). Timing and delay in children vaccination; evaluation of expanded program of immunization in outskirt of Iranian cities. J Res Health Sci.

[10] Duncan C (2016). Measles mumps and rubella virus vaccine. Reactions.

[11] Esna-Ashari F, Mirchamcy H, Shafiei A, Shamsi-Shahrabadi M, Sabiry G, Sasani A (2002). humoral immune response to mumps (rs-12 strain) vaccine of children 1-7 years old. Arch Razi Inst.

[12] Cusi MG, Correale P, Valassina M, Sabatino M, Valensin PE, Donati M (2001). Comparative study of the immune response in mice immunized with four live attenuated strains of mumps virus by intranasal or intramuscular route. Arch Virol.

[13] Shokri S, Shahkarami M, Shafyi A, Mohammadi A, Esna-ashari F, Hamta A (2013). Evaluation of Seroconversion in Guinea Pigs Following Inoculation of New Formulations of Rubella Vaccine. Iran J Virol.

[14] Shramek G, Deinhardt F (1969). Development of an attenuated mumps virus vaccine II Immune response of animals to vaccination with inactivated and live attenuated mumps viruses. J Immunol.

[15] Marth E, Kleinhappl B (2001). Albumin is a necessary stabilizer of TBE-vaccine to avoid fever in children after vaccination. Vaccine.

[16] Wiedmann RT, Reisinger KS, Hartzel J, Malacaman E, Senders SD, Giacoletti KE (2015). MMR^®^ II manufactured using recombinant human albumin (rHA) and MMR^®^ II manufactured using human serum albumin (HSA) exhibit similar safety and immunogenicity profiles when administered as a 2-dose regimen to healthy children. Vaccine.

